# Professional virtue of civility and the responsibilities of medical educators and academic leaders

**DOI:** 10.1136/jme-2022-108735

**Published:** 2023-03-08

**Authors:** Laurence B McCullough, John Coverdale, Frank A Chervenak

**Affiliations:** 1 Obstetrics and Gynecology, Zucker School of Medicine at Hofstra/Northwell, Hempstead, and Lenox Hill Hospital, New York, New York, USA; 2 Menninger Department of Psychiatry and Behavioral Sciences and Center for Ethics, Baylor College of Medicine, Houston, Texas, USA

**Keywords:** Education, Ethics, Philosophy

## Abstract

Incivility among physicians, between physicians and learners, and between physicians and nurses or other healthcare professionals has become commonplace. If allowed to continue unchecked by academic leaders and medical educators, incivility can cause personal psychological injury and seriously damage organisational culture. As such, incivility is a potent threat to professionalism. This paper uniquely draws on the history of professional ethics in medicine to provide a historically based, philosophical account of the professional virtue of civility. We use a two-step method of ethical reasoning, namely ethical analysis informed by pertinent prior work, followed by identifying the implications of clearly articulated ethical concepts, to meet these goals. The professional virtue of civility and the related concept of professional etiquette was first described by the English physician-ethicist Thomas Percival (1740–1804). Based on a historically informed philosophical account, we propose that the professional virtue of civility has cognitive, affective, behavioural and social components based on a commitment to excellence in scientific and clinical reasoning. Its practice prevents a dysfunctional organisational culture of incivility and sustains a civility-based organisational culture of professionalism. Medical educators and academic leaders are in a pivotal and powerful position to role model, promote and inculcate the professional virtue of civility as essential to an organisational culture of professionalism. Academic leaders should hold medical educators accountable for discharge of this indispensable professional responsibility.

## Introduction

Incivility among physicians, between physicians and learners, and between physicians and nurses or other healthcare professionals has become commonplace.^1–5^ Incivility can be expressed in a variety of verbal and sometimes physical behaviours, including dismissing the views of others without explanation, repeatedly demeaning others who are subordinate to one’s power, or patterns of disrespect based on minority status. Women may be at greater risk for experiencing incivility compared with men^6^ and those subjected to it can experience fear, stress and humiliation.^7^ Incivility can disrupt interprofessional collaboration^8 9^ and has adversely affected scientific reporting about the COVID-19 pandemic.^10^ These and other negative responses can jeopardise the quality and safety of patient care.^11^ The managerial literature emphasises that incivility, especially if it is allowed to continue unchecked by poor leadership,^12–15^ can seriously damage organisational culture. For example, nursing unit managers or servant leaders who prioritised their followers’ needs above their own amplified positive behaviours and interactions, whereas unit-level virtuous climates were negatively associated with unit-level incivility.^15^ One recent review, however, concluded that there was a dearth of research on virtue ethics and nursing.^16^ Incivility is a potent threat to professionalism. As such, incivility is an unacceptable practice in medicine, and therefore, should be prevented.

Promoting civility is an essential but neglected aspect of the professional responsibilities of medical educators and academic leaders. The professional virtue of civility should be understood to be a component of professionalism. Leaders of academic health centres, therefore, should work with faculty colleagues to create and sustain a civility-based organisational culture of professionalism.^13–15 17^ Organisational culture comprises an organisation’s stated mission and core values, its policies and practices, what its leaders encourage and discourage, and what its leaders tolerate, especially when leaders tolerate what should not be tolerated.^17 18^ Given that incivility should not be tolerated, it is therefore surprising that the concept of civility has not been clearly articulated in its explicitly ethical dimensions as a professional virtue. Efforts to instil civility as a component of professionalism in learners and to improve the organisational cultures of academic health centres require clear conceptual guidance. Civility is also important for optimising the learning environment and for protecting against burnout and other negative emotional states in learners.

In this paper, we, therefore, pursue two goals. First, we provide a historically based and philosophical account of the ethical concepts of civility and incivility in their cognitive, affective, behavioural and social components. We will show that civility is a professional virtue based on the commitment to excellence in scientific and clinical reasoning. Second, we identify the implications of these ethical concepts for the professional responsibility of medical educators and of leaders in academic medicine to prevent dysfunctional organisational cultures of incivility and to sustain an organisational culture of professionalism based on civility.

## Ethical reasoning about civility and incivility

One method for ethical reasoning comprises two steps.^18 19^ The first is ethical analysis, the clearest possible articulation of pertinent concepts. In the current context, these ethical concepts are civility, incivility, organisational culture and professionalism (box 1). The second is to identify the implications of these clearly articulated ethical concepts. Here, we focus on the professional responsibility of medical educators and leaders of academic medical centres to promote an organisational culture of professionalism.

Box 1 **Four key concepts**

Civility is a professional virtue with cognitive, behavioural and social components. Its cognitive component is the belief that excellence in scientific reasoning is an indispensable basis for professional relationships among clinical colleagues, of clinical investigators to human subjects of research and of educators to learners. The affective component of professional virtue of civility is the satisfaction that comes from the lived commitment to excellence in scientific and clinical reasoning. The behavioural component is sustained attention to and critical appraisal of the clinical reasoning and concerns of colleagues and learners. The social component is an organisational culture based on civility resulting in an organisational culture of professionalism created by medical educators with the support of academic leaders.

Incivility abrogates the commitment to scientific and clinical excellence that is essential for social cohesion in a healthcare organisation, and therefore, for the cooperation that is essential for patient quality and safety. Incivility occurs when the behavioural and social components of civility are violated.

Organisational culture comprises an organisation’s express mission and values, its priorities as expressed in policies, practices and budgets, what leadership values and incentivises, what leadership disvalues and disincentivises, and what leadership tolerates, especially when leadership tolerates what should not be tolerated.^17^

Professionalism comprises the sustained commitments to scientific and clinical excellence in clinical care, research and education, and to making the protection and promotion of the health-related interests the physician’s primary concern and motivation, keeping individual and group self-interest systematically secondary.^17^

The first step of ethical reasoning, ethical analysis, should be informed by pertinent, previous work. We, therefore, aimed to identify how the concepts of civility and incivility have been articulated in the context of professional virtues. Two of us (LBM and JC) conducted a purposeful (and therefore not systematic) search of PubMed to identify papers on concepts of civility and incivility related to the healthcare professions using “incivility” in combination with other terms such as “review,” “medical student,” “physician,” “professionalism,” “ethics,” “concept of incivility” and “concept of civility.” For example, “civility” combined with “virtue” mapped as key words in PubMed returned 174 articles. Because legal literature might contain accounts of the concepts of civility and incivility, we also combined “civility” or “incivility” with “professional liability,” “law” and “case law.” Two of us (LBM and JC) also searched Google Scholar, PsycINFO, CINAHL and ERIC databases to identity scholarship on the concepts of civility and incivility in sources not indexed by PubMed. All searches were initially completed by February 2022 and were updated in January 2023.

We were unable to identify any articles that explicitly provided a historically based, philosophically substantive analysis of civility and incivility including practical ethical implications for contemporary medical educators and academic leaders to promote the professional virtue of civility as essential to creating and sustaining an organisational culture of professionalism. We were also unable to identify any citations about the professional liability or legal dimensions of incivility.

One approach to shaping accounts of civility and incivility is to focus on examples of related behaviours. This is inadequate for completing the step of ethical analysis. Plato explains why. In the eponymous Dialogues Plato (c. 427–c. 348 BCE) has Socrates (c.470–399 BCE) press his interlocutors to get beyond behavioural examples of a concept and to explicitly express the content of a concept because, in the absence of such an account, we cannot be confident that examples, say, of piety in Euthyphro, are indeed examples of piety.^20^ We also found articles by the search methods described above that adopted a second approach to shaping accounts of civility and incivility. This was to provide a concept of civility or incivility, although we found different versions of these concepts,^13 21–28^ as illustrated with selected text in box 2. We could find no consistent agreement, therefore, on a single account of the concepts of incivility and civility and therefore the professional virtue of civility.

Box 2 **Selected concepts of civility and incivility**

- Behavioural concepts of civility and incivility

(Incivility is the) ‘low intensity deviant behaviour with ambiguous intent to harm the target, in violation of workplace norms for mutual respect.’ (Andersson LM, Pearson and Pearson, p. 457)^13^

‘Civil behaviour involves treating others with dignity, acting with regard to others’ feelings, and preserving the social norms for mutual respect. Observing formal rules of etiquette has less to do with civility than does being polite and demonstrating a sensibility of concern and regard.’^13^

‘Workplace civility, then-as a behaviour involving politeness and regard for others in the workplace, within workplace norms for respect- …’^13^

‘We have established that incivility involves acting rudely or discourteously, without regard for others, in violation of norms for respect in social interactions. It follows, then, that workplace incivility involves acting with disregard for others in the workplace, in violation of workplace norms for respect. Workplace norms are the norms of the community of which one is a part while at work, consisting of basic moral standards and others that have arisen out of the tradition of that community, including those prescribed by formal and informal organisational policies, rules and procedures.’^13^

‘The attributes of incivility were verbal or non-verbal behaviours that demean, dismiss or exclude the individual.’^21^

‘Ambiguous intent, violation of mutual respect, low intensity and lack of physical assault were identified as the defining attributes of workplace incivility.’^22^

‘Civility is synonymous with courtesy, politeness and relates to custom and civil law. It implies a more considerate or dignified politeness with the attached training in humanities. It is the avoidance of rudeness.’^23^

[Incivility is the) ‘Disruptive behaviour, which we define as behaviour that does not show others an adequate level of respect and causes victims or witnesses to feel threatened …’^24^

- Social concepts of civility and incivility.

(Incivility) ‘includes both subtle and obvious levels of rude and discourteous behaviour.’^25^

(Civility:) ‘comportment, as an attribute of caring, is defined as one’s bearing, demeanour and agreement or harmony with self and others.’^26^

(Incivility is) ‘the violation of established societal norms that call for respect of others in the environment.’^27^

‘To be ‘civil’ is to be polite, respectful and decent. Conversely, ‘incivility’ is defined as speech or action that is discourteous, rude or impolite.’^28^

Given these limitations in the literature, we turned to two invaluable resources for ethical analysis, the history of Western philosophy and the history of professional ethics in medicine. In the history of Western philosophy the concept of a virtue has been articulated as a sustained, valued character trait expressed in ethically obligatory behaviour.^18^ Virtues create formation of character: becoming the kind of person whose virtue-based behaviour is habitual and therefore on which others can rely. For example, in Euthyphro, Plato has Socrates demonstrate to the hapless Euthyphro that, despite his avowed commitment to the virtue of piety, his ‘pious’ behaviour is inconsistent, making him an unreliable person.^20^

Virtues have cognitive, affective, behavioural and social components.^17 29 30^ The cognitive component is a belief. The affective component is the sense of personal and moral satisfaction that one experiences as one acquires and then sustains the requisite habitual behaviour based on that belief. The behavioural component is the set of ethically obligatory behaviours that put the cognitive and affective components into practice. The social dimension is the ability of others to rely on this ethically obligatory behaviour, because, when habitual and grounded in the cognitive and affective components of character, behaviour becomes consistent. The degree of reliability can vary; the more consistent the behaviour and therefore its rootedness in sustained belief and affect, the more that others can count on it to recur in the future as an expression of reliably moral character. The public virtue of civility sustains a sense of social solidarity based on the belief of social equality, which is a familiar contemporary discourse. The locus classicus for human equality was succinctly stated by the Roman African playwright, Terence (d.c. 159 BCE), homo sum humani nihil a me alienum est (I am a human being and nothing human is alien from me).^31^

## Civility in the history of medical ethics

In the history of medical ethics, the English physician-ethicist, Thomas Percival (1740–1804) was the first to argue that the professional virtue of civility and the related ethical concept of professional etiquette are essential for an ethics of cooperation among otherwise competitive clinicians. For Percival, the competition between physicians and surgeons, with their separate Royal Colleges and prerogatives, threatened the quality and safety of patient care in Manchester Royal Infirmary. Percival’s medical ethics, therefore, made the ethics of cooperation a basis for an organisational culture of professionalism.^32^

Building on Percival, we propose the following, historically informed account of the professional virtue of civility in medicine to complete the step of ethical analysis. Its cognitive component is the belief that the commitment to excellence in scientific and clinical reasoning is an indispensable basis for professional relationships among clinical colleagues, of clinical investigators to human subjects of research and of educators to learners. The affective component of the professional virtue of civility is the deep sense of satisfaction that clinicians and learners experience when they live out this commitment in daily clinical practice and learning. The behavioural component is sustained attention to and critical appraisal of the clinical reasoning and concerns of colleagues and learners. The social component is an organisational culture based on civility resulting in an organisational culture of professionalism created by medical educators with the support of academic leaders.

The professional virtue of civility generates mutual respect. On this account, mutual respect becomes the product of the shared commitment to scientific and clinical excellence that is essential for cooperation in patient care, research and education. This civility-based mutual respect also becomes the basis for transcending differences of social background, gender, race and ethnicity, as well as differences in seniority in the medical and pedagogical hierarchies. An important additional historical origin of mutual respect is Immanuel Kant’s (1724–1804) admonition of respect for persons as having intrinsic value for themselves and for all others.^33^

Percival was especially concerned about due regard for the working sick poor who made up the patient population at Manchester Infirmary and who were at risk of class-based disrespect.^32^ Percival also emphasised that the professional virtue of civility requires respect for all colleagues who are committed to scientific and clinical excellence, especially those with whom physicians then competed for market share—other physicians, surgeons and apothecaries. Percival also emphasised civility as a social virtue because society’s cohesion depends on it and because in its absence violent management of differences gains a foothold that it should not be allowed to do.^32^ This explains why Percival excoriates physicians who participated as attending surgeons to those participating in then-still current, although illegal, duelling.^32^

Professional etiquette, which is emphasised in the current literature on incivility (box 2), plays an essential role in Percival’s professional ethics of cooperation. Professional etiquette takes the form of ritualised behaviours that express the cognitive and affective components of the professional virtue of civility. These ritualised behaviours constitute professional courtesy.^34^

Some have suggested that etiquette should be taught.^35^ Percival attempted to do so and appears to be the first to have done so, in that he prescribed an order for discussing clinical issues during rounds. That was that the most junior clinician speaks first, so that the group has the advantage of the most current scientific information. The senior clinician speaks last, drawing on all that has been said and drawing on greater clinical experience in making a diagnosis and treatment plan. This order of presentation also serves to prevent enthusiasm (a belief in clinical benefit without evidence), the risk of which is higher for the more junior clinicians.^32^ Percival’s etiquette of teaching rounds has lost none of its currency.

The current literature on incivility does not take account of what we call counterfeit civility, a scholarly shortcoming that our historically informed approach corrects. Percival and his contemporaries were familiar with and troubled by the phenomenon of what they called the ‘man of false manners’.^31 32^ The behavioural component of civility can exist without the cognitive and affective components, resulting in counterfeit civility or the surface appearance of civility which can be misleading about true character. Counterfeit civility breeds a cynicism that undermines mutual respect. Observable behaviour with clinical colleagues, learners, and patients and their families that is not consistently respectful is one sign of counterfeit civility. Observed private behaviour may be even worse.

Our account of the relationship between the professional virtue of civility and mutual respect explains why the current literature on incivility emphasises the importance of mutual respect (box 2). Incivility is the absence of all four components of the professional virtue of civility and therefore abrogates the mutual respect that is essential for social cohesion and therefore for the cooperation that is essential for patient quality and safety. When there is a pattern of violation of the behavioural and social components, the hypothesis that an individual lacks the cognitive and affective components of civility becomes plausible. Bullying, harassment, sexual predation, racist behaviour or belittling learners, especially women and learners of colour, should be understood as evidence of the complete absence of the cognitive, affective, behavioural and social components of civility.

## The role of medical educators and academic leaders

Medical educators should teach the concept of the professional virtue of civility (box 1). In this teaching, educators should invite learners to describe behaviours that they have observed and that they consider to be civil or uncivil and explain why this judgement is warranted. Faculty should make clear that learners can come to them with their concerns about incivility and that these will be appropriately addressed. Such teaching becomes an important component of making the discourse of the professional virtue of civility a key component of medical education.

Medical educators should never engage in incivility and should instead be consistent role models of civility, to emphasise that the professional virtue of civility is seamless and transferable to all patient care settings, especially to patients themselves and their family members. Percival’s work updated to a contemporary context means that the professional virtue of civility requires respect for all patients, especially those who come from socioeconomic circumstances that may differ from those of clinicians and learners and those whose first language, habits, mannerisms, personal beliefs and social expectations may also differ. The key to implementing the professional virtue of civility is to treat all patients with respect.

Here, Darwall’s concept of ‘recognition respect’ is helpful.^36^ Recognition respect means that everyone is ‘entitled to have other persons take seriously and weigh appropriately the fact that they are persons in deliberating about what to do’. (Darwall, p.38)^36^ For example, to show respect in this sense and thereby to promote professional identify formation, patients should be asked for permission to use their first names, which can be a sensitive issue for female patients and for patients of colour who have experienced the use of first names without permission as a form of social aggression or invidious discrimination. Medical educators should explain the importance of this and other forms of respect for patients to learners and support them in showing respect for patients. Civility also creates the ethical obligation of medical educators to learners to encourage them to speak up without fear of being demeaned or dismissed for a relative lack of knowledge or clinical experience. The goal should be to create civility-based safe and supportive learning environments that will help to prevent unnecessary learner stress and anxiety.

Medical educators should not tolerate incivility when it occurs, because tolerance encourages unprofessional attitudes and behaviour. Corrective action should be prompt and unambiguous in the message that incivility is never acceptable. Learners should be encouraged to report incivility to supervising faculty or administrative superiors when incivility is exhibited by a faculty member or by a learner, and learners should be protected from retribution or retaliation when they make such reports. Medical educators are already familiar with this non-punitive approach from their training in creating a culture of patient safety.

There can be personal risk in confronting someone who has been uncivil, especially but not exclusively when that colleague holds a position of power, for example, as senior faculty or as division chief or even chairperson, or influence, for example, from having extramural research support or being a ‘rainmaker’ who generates many admissions and referrals. Sir William Osler, in his justly famous essay, Aequanimitas, provides pertinent guidance: ‘You cannot hope, of course, to escape from the cares and anxieties incident to professional life. Stand up bravely, even against the worst.’(Osler, p. 8)^37^ Medical educators should advocate for organisational policies that protect faculty members when they seek to report or correct incivility by colleagues. Such policies are essential for promoting the culture of mutual respect that defines civility.

Academic leaders also have an essential role to play in creating and sustaining a civility-based organisational culture of professionalism. The first step in promoting any professional virtue is to prioritise the virtue as an essential component of organisational culture.^14^ Statements of core values should therefore include civility. An organisational culture that explicitly values civility should be seen as essential for achieving organisational goals regarding equity, diversity and inclusion because incivility is antithetical to achieving the mutual respect that is essential for achieving these valued institutional goals. Creating an organisational culture of professionalism includes preparing orientation and training materials for all members of an organisation, especially educators and learners, which elaborate on the four components of civility.

Percival’s etiquette of teaching rounds was designed so that senior clinicians learnt from their junior colleagues. To update Percival, senior faculty should display the habit of being open to alternative ideas, including from junior colleagues and, importantly, learners at all levels, that pass muster in critical appraisal. Learners should be taught and supported to meet such standards. Teaching critical appraisal and its etiquette of Percivalian mutual respect thus becomes an essential component of professional formation in civility. The etiquette of critical appraisal requires educators to display a willingness to reconsider clinical judgement based on new ideas and to be open to new ideas with a reliable evidence base. Dogmatic or belittling responses to the new ideas of learners or colleagues in other healthcare professions should be understood as manifestations of incivility that are ‘never events’ from the perspective of the professional virtue of civility The social component is the resulting civility-based organisational culture of professionalism. An important aspect of the social component is that promoting civility may help to prevent burnout by sustaining justified self-esteem that occurs when individuals experience being valued members of the clinical, research or learning team.

The professional virtue of civility should be seen as supporting consistent attitudes of due regard and behaviours that express due regard across all professional settings. Clinical faculty should be incentivised to become role models of this professional consistency, in how they treat clinical colleagues, learners and administrators. Academic leaders should create formal systems to recognise and reward exemplary role modelling of civility.

An important corollary is that incivility should be understood by everyone as incompatible with civility and therefore impermissible. There should, therefore, be safe pathways for reporting incivility in all its forms and that are, and are also seen to be, fair. A crucial component of organisational culture occurs when leadership tolerates what should not be tolerated. To borrow from the discourse of patient safety, academic leaders should establish policies that define and treat instances of incivility as never events. Learners, including medical students, have an especially important role to play in activating these pathways and being protected when they do so fairly.^38^ Part of their professional formation is learning how to use such pathways responsibly, which will serve them well in sustaining safety cultures in their clinical practices.

The professional virtue of civility should guide educators, learners and academic leaders in the responsible management of disagreement. There is room for controversy but only when it is evidence-based about scientific and clinical topics and argument-based about ethics topics.^19^ Mere controversy can quickly become personal and even toxic, an approach ruled out by a civility-based organisational culture of professionalism. Civility requires evidence-based and argument-based reasoning as antidotes to incivility that provide standards for responsibly managing disagreement and controversy. In this way, the professional virtue of civility supports the important self-assessment tools of mindfulness and reflective practice.

Academic leaders must be attentive to the hidden curriculum in all its respects. There can be a hidden curriculum and culture of incivility that is toxic to professional formation and therefore to an organisational culture of professionalism. An inability of leaders to recognise, acknowledge and confront incivility may perpetuate a culture of silence and further institutionalise the hidden curriculum.^12^ Leaders should not tolerate a hidden curriculum of incivility and respond rapidly and effectively with a policy of zero tolerance and effective prevention.

## Conclusion

We have shown how ethical reasoning about the professional virtue of civility can be uniquely elucidated by appealing to the history of Western philosophy and to the history of professional ethics in medicine. Our approach to incivility is therefore distinctive for its reliance on the work of historical figures, especially Percival’s professional ethics in medicine. Incivility should be treated as never permissible in clinical practice, teaching, research and administration. Medical educators and academic leaders should not hesitate to use their pivotal and powerful positions to role model, promote and inculcate professional civility as essential to an organisational culture of professionalism and therefore to institutional goals of equity, diversity and inclusion. Academic leaders should hold medical educators accountable for discharge of their indispensable professional responsibility to promote civility as a defining characteristic of an organisational culture of professionalism in medicine.

## Data Availability

No data are available. Not applicable.
